# Influence of image analysis strategy, cooling rate, and sample volume on apparent protein cloud-point temperature determination

**DOI:** 10.1007/s00449-020-02465-8

**Published:** 2020-11-25

**Authors:** Marieke E. Klijn, Jürgen Hubbuch

**Affiliations:** Institute of Engineering in Life Sciences, Section IV: Biomolecular Separation Engineering, Karlsruhe Institute of Technology (KIT), Fritz-Haber-Weg 2, 76131 Karlsruhe, Germany

**Keywords:** Freezing, Protein stability, Colloidal stability, Nucleation temperature, High-throughput screening, Liquid–liquid phase separation

## Abstract

The protein cloud-point temperature (*T*_Cloud_) is a known representative of protein–protein interaction strength and provides valuable information during the development and characterization of protein-based products, such as biopharmaceutics. A high-throughput low volume *T*_Cloud_ detection method was introduced in preceding work, where it was concluded that the extracted value is an apparent *T*_Cloud_ (*T*_Cloud,app_). As an understanding of the apparent nature is imperative to facilitate inter-study data comparability, the current work was performed to systematically evaluate the influence of 3 image analysis strategies and 2 experimental parameters (sample volume and cooling rate) on *T*_Cloud,app_ detection of lysozyme. Different image analysis strategies showed that *T*_Cloud,app_ is detectable by means of total pixel intensity difference and the total number of white pixels, but the latter is also able to extract the ice nucleation temperature. Experimental parameter variation showed a *T*_Cloud,app_ depression for increasing cooling rates (0.1–0.5 °C/min), and larger sample volumes (5–24 μL). Exploratory thermographic data indicated this resulted from a temperature discrepancy between the measured temperature by the cryogenic device and the actual sample temperature. Literature validation confirmed that the discrepancy does not affect the relative inter-study comparability of the samples, regardless of the image analysis strategy or experimental parameters. Additionally, high measurement precision was demonstrated, as *T*_Cloud,app_ changes were detectable down to a sample volume of only 5 μL and for 0.1 °C/min cooling rate increments. This work explains the apparent nature of the *T*_Cloud_ detection method, showcases its detection precision, and broadens the applicability of the experimental setup.

**Electronic supplementary material:**

The online version of this article (10.1007/s00449-020-02465-8) contains supplementary material, which is available to authorized users.

## Introduction

Quantification of protein–protein interactions can be used to assess the colloidal stability of biopharmaceutical formulations [1] or to identify environmental conditions which induce desired phase transitions for separation techniques [2] and 3-D structure determination of proteins [3]. One of the empirical parameters to quantify protein–protein interactions is the protein cloud-point temperature (*T*_Cloud_) [4]. This temperature represents the point at which a protein solution displays liquid–liquid phase separation (LLPS). The temperature at which LLPS is detected is mainly determined by protein–protein interaction strength, where higher *T*_Cloud_ values are found for strong attractive forces and low *T*_Cloud_ values for weaker attractive forces [5]. The accompanying increase in solution turbidity when LLPS occurs is a characteristic that is often exploited for *T*_Cloud_ measurements by means of spectroscopic methods [6, 7], light scattering detection [5, 8–13], or imaging and microscopy [14–18].

Preceding work presented a novel experimental setup to detect *T*_Cloud_ in a high-throughput manner (60 samples per experiment) using a low sample volume (24 μL) [18]. To cover a wide range of protein–protein interaction strengths, the experimental setup included a cryogenic device. This allowed for *T*_Cloud_ detection at sub-zero temperatures in addition to above zero temperatures. Despite the confirmed robustness of the developed method, a comparison with literature data obtained with light scattering showed that only an apparent *T*_Cloud_ (*T*_Cloud,app_) is detectable with the developed experimental setup, as normalized values showed similar trends but the absolute *T*_Cloud_ values were different. Thus, the results obtained with the developed method were found to be robust and precise, but the apparent nature of *T*_Cloud_ rendered the absolute values meaningless for inter-study comparison.

To ensure the usability of historic and future *T*_Cloud_ data across the field to further elucidate protein phase behavior, it is of importance to understand *T*_Cloud,app_ and how the employed experimental setup influences its apparentness. Previously proposed sources that cause *T*_Cloud,app_ were the differences in cooling rate, sample volume, and the employed detection approach. Due to the limited information available in the literature to fully evaluate the influence of these experimental parameters on *T*_Cloud_ detection with any experimental setup, a conclusive reason for the observed apparentness is lacking. The work presented in this study aims to identify the source of the observed *T*_Cloud,app_.

The combination of a cryogenic device and an automatic image acquisition system allows for monitoring the protein solution behavior at sub-zero degrees Celsius, which was applied for the quantification of (weakly) attractive protein–protein interactions, represented by sub-zero *T*_Cloud_ values. Various image processing strategies are available to highlight image objects or image characteristics for image analysis purposes, such as segmentation, defocusing, and smoothing [19]. Work reported by other research groups that employ visible light-based methodologies to determine *T*_Cloud_ extract the mean gray level (MGL) [17], light intensity [14], or manually determine clouding by means of microscopy [15, 16]. The methodology developed in our lab employs the total intensity difference (TID) of images, where each image is compared to the starting image to detect change over time [18]. The current work investigates the effects of different visible light image analysis strategies and determines whether differences in absolute *T*_Cloud,app_ values occur as a result of the analysis strategies. In addition, other temperature points of interest may arise when protein solutions are cooled to sub-zero temperatures, such as the solution freeze point, the solution thaw point, or the solutions’ ice nucleation temperature. Knowledge and understanding of these temperature points can be applied in protein formulation research, as it aids the design of optimal storage procedures for frozen or freeze-dried products [20–23]. Such temperature points may already be present in the captured image-based data but currently applied image analysis strategies are not able to extract this information.

In the scope of this research, three image analysis strategies were used to investigate the effects on *T*_Cloud,app_ determination and to explore a broader application of the developed experimental setup. This included two previously reported image analysis strategies for *T*_Cloud_ detection, namely MGL [17] and TID [18]. As the camera used in this work captures color images, the mean red, blue, and green color pixel values were explored in addition to the gray color level. The third image analysis strategy was based on binarized images, where images are processed to solely contain black and white pixels using different threshold values.

The experimental parameters which were investigated during this study are the cooling rate and sample volume. The cooling rate was varied between 0.1 °C/min and 0.5 °C/min, with increments of 0.1 °C/min, where 0.1 °C/min is the slowest cooling rate of the employed cryogenic device as well as the smallest step size. This relatively narrow range was selected to investigate whether small cooling rate increments already affect the *T*_Cloud,app_ absolute value and, in parallel, to provide information on the detection sensitivity of the experimental setup. In addition, a maximum of 0.5 °C/min was chosen, as faster cooling rates may lead to deviating temperatures between the set and measured temperature during the course of an experiment. The sample volume was varied between 5 μL and 24 μL, where 24 μL was the original sample volume described in the preceding work. A reduction in sample volume was preferred to capture not only the effects of sample volume changes but also to further miniaturize the experimental setup, which is a beneficial characteristic for high-throughput screening applications due to reduced material usage.

To summarize, the work presented in this study includes the investigation of three different image analysis strategies and two experimental parameters. It was aimed to understand the source of *T*_Cloud,app_ and explore the information content of the generated data beyond *T*_Cloud_ detection.

## Materials and methods

### Buffer preparation

A 0.6 molar sodium phosphate buffer at 8 different pH values (5.8, 6.0, 6.3, 6.5, 6.8, 7.2, 7.4, and 7.8) was employed. The buffer components were sodium di-hydrogen phosphate (monohydrate; Merck KGaA, Darmstadt, Germany) and di-sodium hydrogen phosphate (anhydrous; VWR, Radnor, PA, USA). After preparation, and after two weeks of storage, the buffer solutions were filtered over a 0.2 µm Supor®-200 filter (Pall Corporations, Port Washington, NY, USA). This buffer system was chosen to use literature data provided by Taratuta et al. [13] as a validation set. The pH value was set with a five-point calibrated pH meter (HI-3220, Hanna® Instruments, Woonsocket, RI, USA), which was equipped with a SenTix® 62 pH electrode (Xylem Inc., White Plains, NY, USA). The pH value of all buffers was checked throughout the study and was maintained within ± 0.05 pH unit of the desired pH values.

### Protein solution preparation

A 90 g/L hen-egg white lysozyme (Hampton Research, Aliso Viejo, CA, USA) solution was prepared on the day of experimentation. The required amount of protein was dissolved in the respective buffer. The solution was run over a 0.2 µm Supor® polyethersulfone (PES) pre-syringe filter (Pall Corporations, Port Washington, NY, USA). The protein stock concentration was measured with a NanoDrop™ 2000c UV–Vis spectrophotometer (Thermo Fischer Scientific, Waltham, MA, USA), using an extinction coefficient of 22 Lg^−1^ cm^−1^. To remain within the detection range of the spectrophotometer, 1:10 dilutions were prepared for concentration measurements. All prepared protein solutions were used within 7 h and stored at room temperature until use.

### Cloud-point temperature measurements

#### Image acquisition

The protein cloud-point temperature was determined using the setup as described by Klijn et al. [18]. This setup employs a cryogenic device (EF600M 105, Grant Instruments, Cambridgeshire, UK) and a GoPro Hero4 camera (GoPro Inc., San Mateo, CA, USA) for image acquisition. The following GoPro settings were used to minimize image fluctuations: ProTune white balance was set to 4000 K, ISO limits were set to 200 (minimum and maximum), and EV Comp was set to − 2.0. For varying cooling rates, the number of seconds per image was adjusted to match the images per degrees Celsius as closely as possible. This was done to keep the temperature sampling rate comparable between different cooling rates. In addition, this reduces the amount of data for slower cooling rates as these experiments run for a longer period of time. For example, an experiment running from 15 to − 40 °C with a cooling rate of 0.1 °C/min results in 3300 images for a 10 s per image setting, which translate to an image every 0.02 °C. A 0.5 °C/min cooling rate results in 660 images for a similar setup, which is 0.08 °C per image. By adjusting the frame rate to 30 s per image for a 0.1 °C/min cooling rate, one obtains 1100 images, which translates to 0.05 °C per image. Thus, the temperature sampling rate is more comparable and the amount of data is decreased. However, the employed GoPro Hero4 solely allows a setting of 1, 5, 10, 20, 30, or 60 s per image, which is why an exact temperature sampling match could not be realized. The employed seconds per image for a cooling rate of 0.1 °C/min, 0.2 °C/min, 0.3 °C/min, 0.4 °C/min, and 0.5 °C/min, were 30, 10, 10, 10, and 10, respectively. This translates to an image every 0.05 °C, 0.03 °C, 0.05 °C, 0.07 °C, and 0.08 °C, respectively.

#### Experimental parameters

During *T*_Cloud,app_ measurements, the temperature was set to decrease from 15 °C to − 40 °C. An overview of the employed parameters per experiment can be found in Table 1. After a holding time of 1 min at − 40 °C, a heating rate of 3 °C/min was used to return to a temperature of 20 °C. Heating was not monitored as it was shown in previous work that *T*_Cloud,app_ detection with the employed setup is more reliable during cooling than during heating [18]. All samples were prepared in 96-well crystallization plates (MRC Under Oil 96 Well, SWISSCI AG, Neuheim, CH) and sealed with Duck® Brand HD Clear sealing tape (ShurTech® Brands, LC., Avon, OH, USA) prior to measurements to prevent evaporation. All samples were measured as six technical replicates, with which the median (as a measure of central tendency) and median absolute deviation (as measure of spread) of *T*_Cloud,app_ were calculated. Calculation of the median and median absolute deviation *T*_Cloud,app_ was performed after outlier detection. A data point was considered an outlier based on Tukey’s fences [24], employing a whisker length of 0.75 times the interquartile range. For comparison to literature data, the median protein cloud-point temperatures were normalized by means of min–max normalization.

**Table 1:** Overview of employed parameters per experiment

Experimental parameter	Volumes	Cooling rate	Protein concentration	Image analysis strategy	
	(µL)	(°C/min)	(g/L)		
Volume	24, 20, 15, 10, 5	0.5	90	TID	
Cooling rate	24	0.1, 0.2, 0.3, 0.4, 0.5	90	TID	
Image analysis	20	0.5	90	TID TWP MGL	MRL MBL MGrL

*TID* Total intensity difference, TWP Total white pixels (0.50, 0.75, 0.85, *graythresh*, mean intensity), MGL Mean gray level, MRL Mean red level, MBL Mean blue level, MGrL Mean green level

### Data evaluation

All data processing was established in the MATLAB programming environment (version R2018b, The MathWorks Inc., Natick, MA, USA). Data smoothing was performed with a moving mean window of 5% of the total number of data points. Due to insufficient data quality, the experiment with a cooling rate of 0.3 °C/min and a sample volume of 24 µL was smoothed with a moving mean window of 2.5%. Different image analysis strategies were applied to this work. The image analysis strategy referred to as total intensity difference (TID), indicates that image analysis was performed as described in Klijn et al. [18]. In addition to TID, two other strategies were employed, namely the mean color level and the total white pixel count, which will be explained in the following sections.

#### Mean color levels

The mean color value was defined as the mean pixel value of the cropped well image. This value was extracted for each color (red, green, and blue) from the raw color image and the gray image (obtained with the MATLAB function *rgb2gray*). This was done to evaluate the applicability of the image analysis approach presented in other work, where the mean gray value of images was used to detect *T*_Cloud_[17].

#### Total white pixel count

To obtain the number of white pixels in an image, each cropped well image was transformed into a binary image from which the total sum of white pixels was calculated. Conversion of a raw color image to a binary image requires the definition of a white level. The white level determines which pixels will become white in the binary image, thereby influencing the total white pixel count. Five different white levels were investigated, three arbitrarily predefined levels (0.50, 0.75, and 0.85) and two data-dependent levels that were set per microtiter plate well image. The two data-dependent levels included (1) a white level calculated with MATLAB function *graythres* (employing Otsu’s method to find the optimal separation value [25]) based on the first image of each well (*t*_0_) and (2) a white level set to the mean intensity of the gray image of the first image of each well (*t*_0_). Thus, the data-dependent white levels are defined for each well customarily, while the predefined arbitrary levels are set for all wells equally. Two variables were extracted from the obtained data, namely *T*_Cloud,app_ and the ice nucleation temperature (*T*_Nuc_). *T*_Cloud,app_ was defined as the minimum number of total white pixels, which is comparable to the TID definition, and *T*_Nuc_ was defined as the maximum gradient where the total white pixel count increased.

### Thermography

A UV-Star® µClear® half-area 96-well plate (Greiner Bio-One, Kremsmünster, AU) was used to perform a single run on the cryogenic device which was monitored with a thermographic camera (FLIR Ax5, FLIR Systems, Wilsonville, OR, USA). A framerate of 25 frames per second was used and temperature data were extracted with the software FLIR Tools (FLIR Systems, Wilsonville, OR, USA). The measurement was conducted with demineralized water (50 µL) and the plate was sealed with Duck® Brand HD Clear sealing tape. The temperature gradient was set to run from 20 to − 20 °C, with a holding time of 2 min every 5 °C. The thermographic camera was not calibrated or optimized for the atmospheric settings. Therefore, only the relative temperature change from the starting point (*t*_0_) was determined. This allowed for the comparison of the thermographic data with temperature data extracted from the cryogenic device software.

## Results and discussion

### Image analysis strategies

**Fig. 1:**
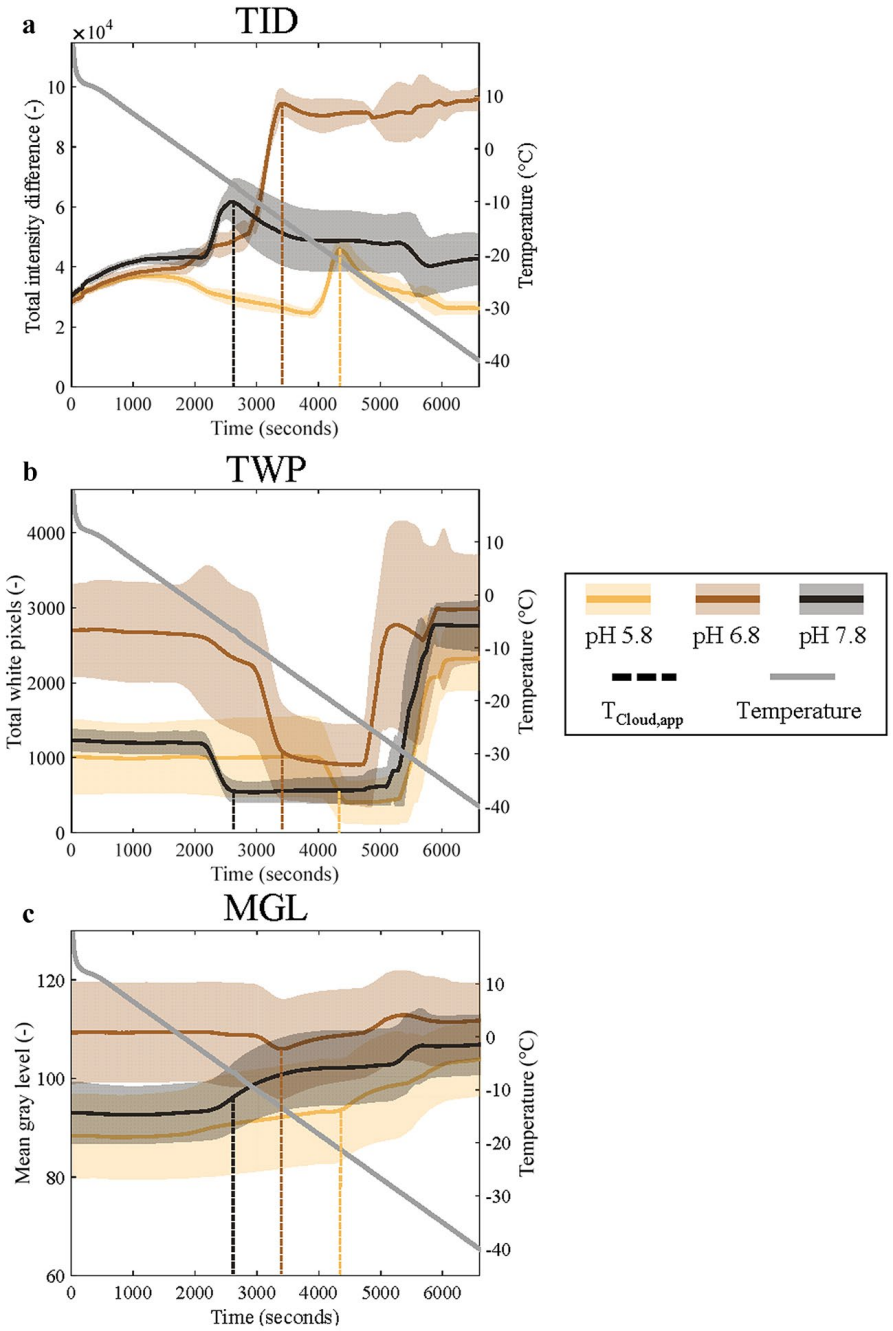
Exemplary results for **a** total intensity difference (TID), **b** total white pixels (TWP) using a white level of 0.50, and **c** mean gray level (MGL) on all left y-axes over time (seconds; *x* axis). The temperature (°C; gray) during the measurement is shown on the right *y* axis. Each plot shows data for samples at pH 5.8 (yellow), pH 6.8 (brown), and pH 7.8 (gray). The correspondingly colored solid lines indicate the median value and the shades indicate the median absolute deviation of 6 technical replicates, respectively. The correspondingly colored vertical dashed lines indicate the position of the apparent protein cloud-point temperature (*T*_Cloud,app_) as obtained with (**a**) to highlight the position in **b** and **c**. The lines are colored per corresponding pH value. All data were obtained with a cooling rate of 0.5 °C/min and a sample volume of 20 μL (color figure online)

To evaluate the influence of different image analysis strategies on *T*_Cloud,app_ detection, three image analysis strategies were performed using the same data set. The image analysis strategies include the TID, the total number of white pixels (TWP), and the evaluation of the mean gray, red, blue, and green level (MGL, MRL, MBL, and MGrL, respectively). Figure 1 shows exemplary data for TID, TWP (using a white level of 0.50), and MGL using data of three samples, namely at pH 5.8, pH 6.8, and pH 7.8 with a sample volume of 20 µL and a cooling rate of 0.5 °C/min. Here, the left *y* axis indicates the respective variable and the right axis indicates the measured temperature by the cryogenic device during the measurement.

#### Total intensity difference (TID)

The TID was extensively discussed in previous work [18] and will therefore be only briefly described here. The TID is a measure of dissimilarity between two images, which increases when a solution moves from transparent to turbid. The relatively sharp increase in TID observed in Fig. 1a as the temperature decreases was identified as the *T*_Cloud,app_. More specifically, *T*_Cloud,app_ was defined as the temperature at which the sharp increase flattened, which represents the point at which solution clouding is concluded. This was observed in the data shown in Fig. 1a at approximately *t* = 2600 s for the sample with pH value 7.8 (black), at *t* = 3200 s for pH 6.8 (brown), and at *t* = 4200 for pH 5.8 (yellow).

#### Total number of white pixels (TWP)

The TWP analysis requires a definition of the white level to separate black and white pixels in each image. In this work, five different white level definitions were evaluated, namely 0.50, 0.75, 0.85, and two data-dependent levels (MATLAB function *graythres* value and mean gray intensity based on the first cropped well image). Figure 1b only shows three exemplary results for data obtained with the 0.50 white level, as this level was found to include two distinct signal changes and the results obtained with other white levels did not result in any significant signal changes or any additional information. Data for the exemplary samples obtained with the other white levels can be found in the Supplementary Material, Figure S1. In addition, to visualize the lack of information available in the images obtained by the other white levels, exemplary images at 15 °C, *T*_Cloud,app_, and − 40 °C can be found in the Supplementary Material in Figure S2.

The first significant signal change in Fig. 1b, represented by a decrease in TWP, corresponds to the increase in TID data seen in Fig. 1a at approximately *t* = 2600, 3200, and 4200 s for pH 7.8, 6.8, and 5.8, respectively (indicated by the vertical line in the corresponding color). This means that *T*_Cloud,app_ is also detected by means of counting white pixels in a binary image. Upon visual inspection of images, it was found that the TWP decrease is caused by a decrease in light reflection by the sample as the sample becomes turbid. In addition to the decrease in TWP at *T*_Cloud,app_, a distinct increase in TWP is seen at a later time point, as the temperature further decreases. Here, a sharp increase in TWP is observed at around *t* = 5000 for all pH values. Based on data obtained for demineralized water samples included in each data series, the observed TWP increase was attributed to the ice nucleation temperature (*T*_Nuc_) of the supercooled samples. The average *T*_Nuc_ of these water samples was − 30.8 ± 2.2 °C (see Supplementary Material, Table S1). The average *T*_Nuc_ is in line with literature values of supercooled water droplets, where T_Nuc_ values down to -32 °C were reported for droplets with a diameter of 1000 µm [26]. For reference, the sample volume employed in this work would roughly translate to a droplet diameter of 2000–5000 μm, assuming a perfect sphere. Even though a *T*_Nuc_ was identifiable with TWP data using a 0.50 white level, a trend for *T*_Nuc_ values was not found among the measured protein samples (see Supplementary Material, Fig. S3). Thus, it is concluded that the sample volume and cooling rate applied in this study do not affect *T*_Nuc_ using the current setup. Notably, *T*_Nuc_ of a sample containing protein, or solely the buffer solution (blank), was identified by means of a TWP increase, while water samples showed a decrease in white pixels upon freezing. An example of these opposite trends can be found in the Supplementary Material, Fig. S4. Visual image inspection showed that the TWP increase for protein samples was due to the formation of white-colored ice, while water samples showed gray ice formation, a color comparable to the turbidity seen during protein solution clouding.

#### Mean color levels

Mean color levels were included in this study, as work by Pincemaille et al. applied extracted MGL to determine *T*_Cloud_[17]. Figure 1c shows exemplary MGL data over the course of a measurement with the setup developed in our lab. The vertical dashed lines in Fig. 1c show the position of *T*_Cloud,app_ as detected in Fig. 1a. The corresponding observed signal changes in Fig. 1c are a slight increase in MGL for pH 7.8 (black) and two slight minima for pH 5.8 (yellow) and pH 6.8 (brown). These inconsistent changes in MGL have an absolute value of approximately 1–10 (depending on the pH value), while Pincemaille et al. reported absolute changes of the MGL with a value of 100. Thus, in contrast to the results presented by Pincemaille et al., the MGL data obtained in this work did not contain enough discriminatory information for the extraction of a consistent signal change that corresponded to *T*_Cloud,app_. All other mean color levels (red, green, and blue) also did not contain discriminatory information. Examples for the red, blue, and green mean color level can be found in the Supplementary Material, Figure S5. Exemplary images for each color level at 15 °C, *T*_Cloud,app_, and − 40 °C can be found in the Supplementary Material as well, in Figure S2. The inability to transfer the MGL image analysis protocol as described by Pincemaille et al. to another camera-based setup illustrates a potential general hardware dependency of image analysis strategies to detect *T*_Cloud_.

#### Validation

**Fig. 2:**
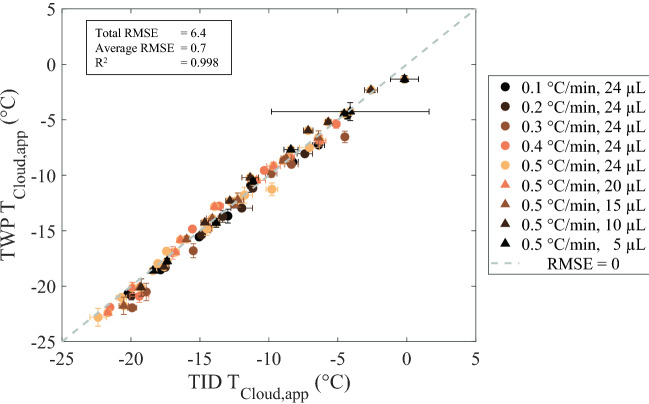
Median apparent protein cloud temperature (*T*_Cloud,app_ in °C) based on the total number of white pixels (TWP; *y* axis) using a white level of 0.50 and the *T*_Cloud,app_ based on the total intensity difference (TID; *x* axis). Circles indicate results obtained for different cooling rates (0.1–0.5 °C/min) and triangles indicate results obtained for different sample volumes (24–5 μL). Darker colors indicate slower cooling rates and lower sample volumes. The dashed line indicates a root mean squared error (RMSE) of 0. The textbox lists the total and average RMSE (°C) of all data points and the R^2^

The *T*_Cloud,app_ results obtained with TID and TWP image analysis were compared to each other to quantify their similarity in absolute *T*_Cloud,app_ values. Figure 2 shows *T*_Cloud,app_ values determined with the TID image analysis strategy (*x* axis), and with the TWP image analysis strategy (*y* axis) for all cooling rates, sample volumes, and pH values. All *T*_Cloud,app_ values obtained with the TWP image analysis strategy can be found in the Supplementary Material, Table S3. The total root-mean-squared error (RMSE) of 6.4 °C, the average RMSE of 0.7 °C, and the *R*^2^ of 0.998 indicate there is a relatively small difference between the two image analysis strategies for *T*_Cloud,app_ detection. One-way ANOVA analysis resulted in an *F* value of 0.07 and a *p* value of 0.80, reflecting an insignificant difference between *T*_Cloud,app_ values obtained with the TID and TWP image analysis strategies (see Supplementary Fig. S6). Nevertheless, considering that MGL was not readily transferable between different setups, a collaborative initiative to evaluate the performance of multiple image analysis strategies in combination with different camera-based experimental setups would be of interest to promote the development of robust and transferable image analytical approaches to facilitate relatively simple and high-throughput *T*_Cloud_ detection.

### Experimental parameters

**Fig. 3:**
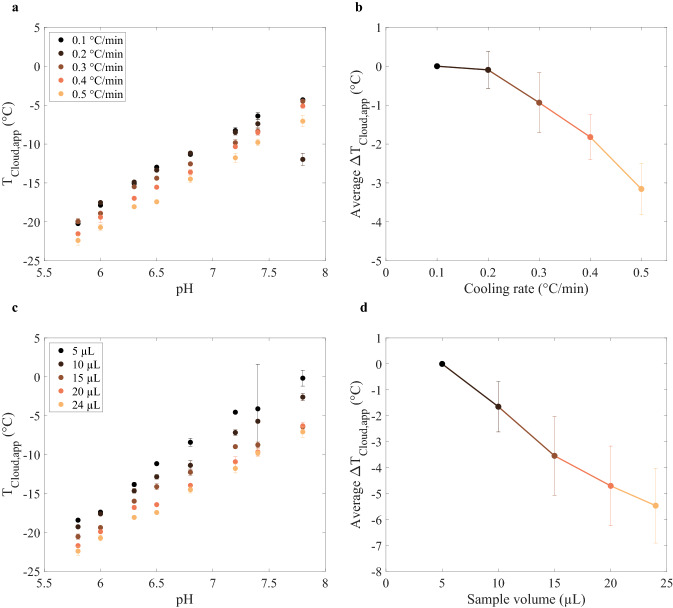
**a** Median apparent protein cloud-point temperature (*T*_Cloud,app_ in °C; *y* axis) per pH value (*x* axis) for varying cooling rates (0.1–0.5 °C/min) and a sample volume of 24 μL, where darker colors indicate slower cooling rates. **b** Average *T*_Cloud,app_ difference (˚C; *y* axis) between *T*_Cloud,app_ obtained with 0.1 °C/min and the data for the respective cooling rate (°C/min; *x* axis), for all pH values. Colors are similar to **a**. **c** Median *T*_Cloud,app_ (°C; *y* axis) per pH value (*x* axis) for varying sample volumes (24–5 μL) and a cooling rate of 0.5 °C/min, where darker colors indicate lower sample volumes. **d** Average *T*_Cloud,app_ difference (°C; *y* axis) between *T*_Cloud,app_ obtained with 5 µL and the data for the respective sample volume (µL; *x* axis), for all pH values. Colors are similar to **c**. For **a** and **c**: the error bars indicate the median absolute deviation. For **b** and **d**: a line was added to guide the eye and the error bars indicate the standard deviation. All data were obtained with the total intensity difference image analysis strategy

In addition to several image analysis strategies, two experimental parameters were varied in this work, namely the cooling rate and sample volume. The cooling rate was varied as previous work showed *T*_Cloud,app_ depression for increasing cooling rate (an increase from 0.5 °C/min to 2.5 °C/min and 10 °C/min [18]). In this work, it was investigated for which cooling rate increment the decrease in *T*_Cloud,app_ could already be detected. The limit and effect of sample volume reduction were investigated as previous work proposed sample volume as a possible factor influencing absolute *T*_Cloud,app_ values. In addition, sample volume reduction allows for evaluating the miniaturization potential, which benefits high-throughput screenings due to a reduced material requirement. The results for both experimental parameters are presented in Fig. 3.

#### Cooling rate

Figure 3a shows the median *T*_Cloud,app_ per pH value for different cooling rates (0.1–0.5 °C/min). All median *T*_Cloud,app_ values can be found in the Supplementary Material, in Table S4. The last data point (pH 7.8) in the 0.2 °C/min cooling rate data series (dark brown) is considered an outlier. This was attributed to a sample preparation error, as a comparable *T*_Cloud,app_ value was found for the data point at pH 6.5 in the same data series. Disregarding this outlier, the results presented in Fig. 3a show that a cooling rate change between 0.1 °C/min and 0.5 °C/min resulted in a consistent decrease of *T*_Cloud,app_ for faster cooling rates. To quantify this observed *T*_Cloud,app_ decrease in Fig. 3a, the average *T*_Cloud,app_ difference between each data point per cooling rate was calculated with respect to the data obtained with a cooling rate of 0.1 °C/min. These average temperature differences are depicted in Fig. 3b, where the error bars indicate the standard deviation of *T*_Cloud,app_ differences obtained with all pH values per data series. Figure 3b indicates that a cooling rate increase from 0.1 °C/min to 0.3 °C/min already results in a reduction of *T*_Cloud,app_ by 0.9 ± 0.8 °C. Overall, an absolute average *T*_Cloud,app_ decrease up to 3.2 ± 0.7 °C was found for increasing the cooling rate from 0.1 °C/min to 0.5 °C/min, respectively. One-way ANOVA analysis resulted in an *F* value of 1.33 an *p* value of 0.27 upon analyzing the data obtained with a cooling rate of 0.1 °C/min and 0.5 °C/min (see Supplementary Fig. S6). This was considered a significant change as it reflects a greater difference in variance compared to the variance obtained between the image analysis strategies (*F* value of 0.07 and *p* value of 0.80) and the high precision of the *T*_Cloud,app_ data (MAD of 0.3 °C for all 40 data points).

#### Sample volume

The resulting median *T*_Cloud,app_ values for sample volumes varying from 5 to 24 μL are presented in Fig. 3c. Here, it is shown that smaller sample volumes led to higher *T*_Cloud,app_ values. The effect of higher *T*_Cloud,app_ values for lower sample volumes was found to be consistent for all pH values. Similar to the cooling rate experiment, the average *T*_Cloud,app_ difference for each sample volume data series was calculated, with respect to the lowest sample volume (5 μL). These differences are depicted in Fig. 3d. A decrease in the *T*_Cloud,app_ value was already detectable for the first sample volume increase from 5 to 10 µL, represented by an average *T*_Cloud,app_ difference of 1.6 ± 1.0 °C. The largest difference was seen between a sample volume of 5 µL and 24 µL, with an average absolute *T*_Cloud,app_ difference of 5.5 ± 1.4 °C. The difference in *T*_Cloud,app_ between a sample volume of 5 μL and 24 μL was considered significant based on ANOVA results, showing a *F* value of 3.33 and *p* value of 0.09 (see Supplementary Material S6). Despite the *T*_Cloud,app_ differences based on sample volume, a detection limit was not reached. Thus, this experiment demonstrated that even a sample volume of as low as 5 μL can be used with the employed experimental setup for a reliable and accurate *T*_Cloud,app_ detection.

#### Validation

**Fig. 4:**
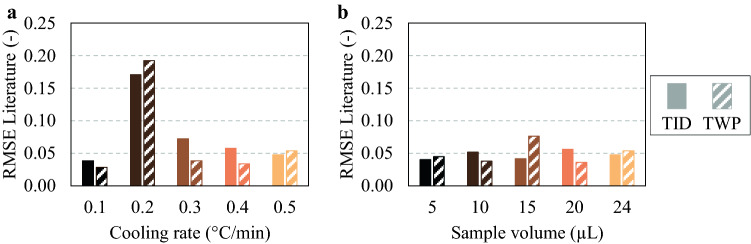
**a** Root mean squared error (RMSE; *y* axis) values for varying cooling rate (°C/min; *x* axis) and **b** varying sample volume (μL; *x* axis). RMSE values were calculated with normalized apparent protein cloud-point temperature values obtained with total intensity difference (TID; solid) and total white pixels using a white level of 0.50 (TWP; hatched), in reference to literature data [13]

*T*_Cloud,app_ values obtained for all cooling rates, and sample volumes were validated by means of comparison to literature data from Taratuta et al. [13]. The RMSE was used to quantify the relative difference between the *T*_Cloud,app_ obtained in each data series, employing both TWP and TID, and literature *T*_Cloud_ data, where low RMSE values indicate a high similarity. The resulting RMSE values for varying cooling rates and sample volumes are presented in Fig. 4a, b, respectively.

With the exception of the data obtained with a cooling rate of 0.2 °C/min, all RMSE values are below 0.08. This is considered a low RMSE value, as all data are normalized between 0 and 1. The high RMSE for 0.2 °C/min is due to the outlier at pH 7.8, as discussed in Sect. 3.2.1. The relatively low RMSE between each data series and literature data confirms that all tested cooling rates, sample volumes, and image analysis strategies result in a similar data variance as a function of the sample pH value. Based on this data, it was concluded that the investigated variables are not the direct source of the apparent nature of *T*_Cloud,app_.

### Thermal imaging

The cooling rate and sample volume experiments showed a *T*_Cloud,app_ depression for faster cooling rates, and higher sample volumes, respectively. Our preceding study speculated that this could be caused by camera-related insensitivity or a difference between the temperature measured by the device and the actual sample temperature [18]. The camera-related insensitivity can no longer be considered a source, as not only the cooling rate but also a sample volume increase resulted in a *T*_Cloud,app_ depression. If camera-related insensitivity occurred, then lower sample volumes would be more difficult to measure with an insensitive detection, and therefore would lead to lower *T*_Cloud.app_ values. As this was not observed, the *T*_Cloud,app_ depression is attributed to the difference between the measured temperature by the cryogenic device and the actual sample temperature during the measurement. To gain insight into the temperature deviations, an exploratory measurement with demineralized water samples was performed, where the visible light camera was substituted with a thermographic camera. It should be noted that using a thermographic camera brings its own technical challenges, as calibration is required for thermographic imaging, measurements are sensitive to atmospheric factors, and new data analytical infrastructure is necessary [27, 28]. Although we are aware of these issues regarding thermographic imaging, optimization of the thermographic setup, and the subsequent data analytical workflow is considered outside the scope of this study. This exploratory experiment was solely used to confirm the existence of a temperature deviation.

**Fig. 5:**
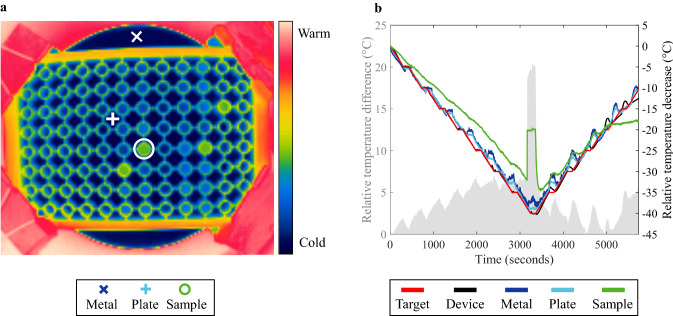
**a** Exemplary thermographic image of 96-well plate. Indicated are the positions of three temperature measurements; metal adapter (cross), microtiter plate (plus), and a sample (circle). The color scale runs from red to blue, indicating high and low temperatures, respectively. **b** Relative temperature decrease (°C; right *y* axis) over time (seconds; *x* axis) set in the controlling software (Target, red), measured by the device during a measurement (Device, black), measured at the metal adapter (Metal, dark blue), measured at the microtiter plate (Plate, light blue), and measured for a demineralized water sample (Sample, green). In **b** the relative temperature difference (°C; left *y* axis) between the sample and the device, measurement is shown in gray (color figure online)

The thermographic results are shown in Fig. 5. A snapshot of the thermographic measurement in Fig. 5a indicates three locations for which temperature data was extracted, namely the metal adapter (Metal, cross), the microtiter plate (Plate, plus), and a demineralized water sample (Sample, circle). In Fig. 5b, the relative temperature decrease (right *y* axis) for these points are plotted over the course of the experiment, alongside temperature data obtained from the cryogenic device, namely the set temperature in the software (Target) and the temperature measured during the experiment (Device). The measurement ran from 20 °C to − 20 °C, with a cooling/heating rate of 1 °C/min and a 2-min holding time every 5 °C. These holding steps were included to determine whether this would benefit temperature control.

The relative temperature decrease was the metric of choice for comparison, as the thermal camera settings were not optimized. Here, the temperatures are relative to the starting temperature of the respective measurement source. To highlight the temperature difference, the left *y* axis in Fig. 5b indicates the difference between the relative sample temperature (green solid line) and the relative device temperature (black solid line). Over the course of the measurement, the sample temperature deviates from the device temperature up to nearly 7 °C after approximately 3250 s (approximately 54 min). The subsequent steep increase in sample temperature (approximately at *t* = 3200 s and a temperature of − 31 °C) is the latent heat of fusion, which is a result of ice nucleation in the supercooled water sample. This confirms the occurrence of *T*_Nuc_ during experimental runs with the employed setup, as was seen in Sect. 3.1.2 for TWP image analysis strategy results.

In addition to the deviation between the sample temperature and the device temperature, a temperature deviation was seen for the metal adapter and the microtiter plate when compared to the device temperature. This indicates that the multi-layered setup as well as the samples are sources of the observed temperature discrepancy. Based on the thermographic results, it can no longer be assumed that the measured temperature by the cryogenic device is equal to the actual sample temperature. Thus, the systematic discrepancy between the measured device temperature and the actual sample temperature is the source of the apparent nature of the detected *T*_Cloud_. The thermographic data shows that a 2-min holding time every 5 °C was not long enough to reach a temperature equilibrium between the device, adapter, plate, and sample for a 24 μL sample volume. Previous studies in literature do not always report on the sample volume and/or the corresponding equilibrium time, but values were found to range from 2 to approximately 30 min for a sample volume of 60–300 μL [6, 17]. Thus, it is recommended that the equilibrium time should be determined separately for different sample volumes, cooling rates, and employed cryogenic device setups. Alternatively, one could determine a *T*_Cloud,app_ correction factor with thermographic sample temperature data, so that one still benefits from high-throughput, low volume, and fast *T*_Cloud_ measurements without time-consuming intermediate equilibrium steps.

## Conclusion

This work was performed to investigate the source of *T*_Cloud,app_ values of lysozyme obtained by means of a cryogenic device in combination with an automated image acquisition system to facilitate a broader understanding of experimental and data evaluation influences and to enable inter-study data comparability. For this, three image analysis strategies, five cooling rates (0.1–0.5 °C/min), and five sample volumes (5, 10, 15, 20, and 24 µL) were investigated in particular. The investigation of different image analysis strategies showed that extraction of the total white pixel count resulted in additional information compared to the previously used total intensity difference, as the total white pixel count is able to detect *T*_Cloud,app_ and *T*_Nuc_. Faster cooling rates showed a decrease in *T*_Cloud,app_, with an average absolute difference of 0.1 ± 0.4 °C up to 3.2 ± 0.7 °C. A similar trend was seen for increasing the sample volume from 5 μL to 24 μL, where the average absolute difference in *T*_Cloud,app_ ranged from 1.6 ± 1.0 °C to 5.5 ± 1.4 °C. The observed *T*_Cloud,app_ was affected by the investigated experimental variables due to a discrepancy between the measured device temperature and the actual sample temperature, which was confirmed based on a thermographic measurement. Despite sample temperature discrepancy, the precision of the employed experimental setup was shown by the ability to detect differences in *T*_Cloud,app_ values induced by small cooling rate increments (0.1 °C/min). Moreover, the sample volume was reduced by 80% from 24 µL down to 5 µL, which still yielded reliable results. This demonstrates that the employed experimental setup is not only capable of precise measurements, but that it is also highly sensitive. Overall, the presented work identified the source *T*_Cloud,app_ as well as the influence of the sample volume and cooling rate in this regard, while simultaneously miniaturizing the setup and indicating a potential application expansion of the employed experimental setup by means of a different image analysis strategy.

## Electronic supplementary material

Below is the link to the electronic supplementary material.

Supplementary material 1 (pdf 1119 kb)
