# Supplementary material for: Influence of image analysis strategy, cooling rate, and sample volume on apparent protein cloud-point temperature determination
Source: Bioprocess Biosyst Eng. 2020 Nov 25;44(3):525–36. doi: 10.1007/s00449-020-02465-8 (PMC7889528; doi:10.1007/s00449-020-02465-8)
Supplement: Supplementary file 1 — Supplementary material 1 (pdf 1119 kb) [file 449_2020_2465_MOESM1_ESM.pdf]

SUPPLEMENTARY MATERIAL TO

INFLUENCE OF IMAGE ANALYSIS STRATEGY, COOLING RATE, AND SAMPLE VOLUME ON APPARENT PROTEIN CLOUD-POINT TEMPERATURE DETERMINATION

Marieke E. Klijn[1], Jürgen Hubbuch[1]*

[1] Institute of Engineering in Life Sciences, Section IV: Biomolecular Separation Engineering, Karlsruhe Institute of Technology (KIT), Fritz-Haber-Weg 2, 76131 Karlsruhe, Germany

* Corresponding author. Tel: +49 721 608-42557; fax: +49 721 608-46240. E-mail address: juergen.hubbuch@kit.edu

Figure S1 shows exemplary data for different white levels, where the information did not contain any distinct signal changes (Figure S2a and Figure S2b) or not more information in addition to a distinct signal change for the apparent protein cloud-point temperature (Figure S2c and Figure S2d).

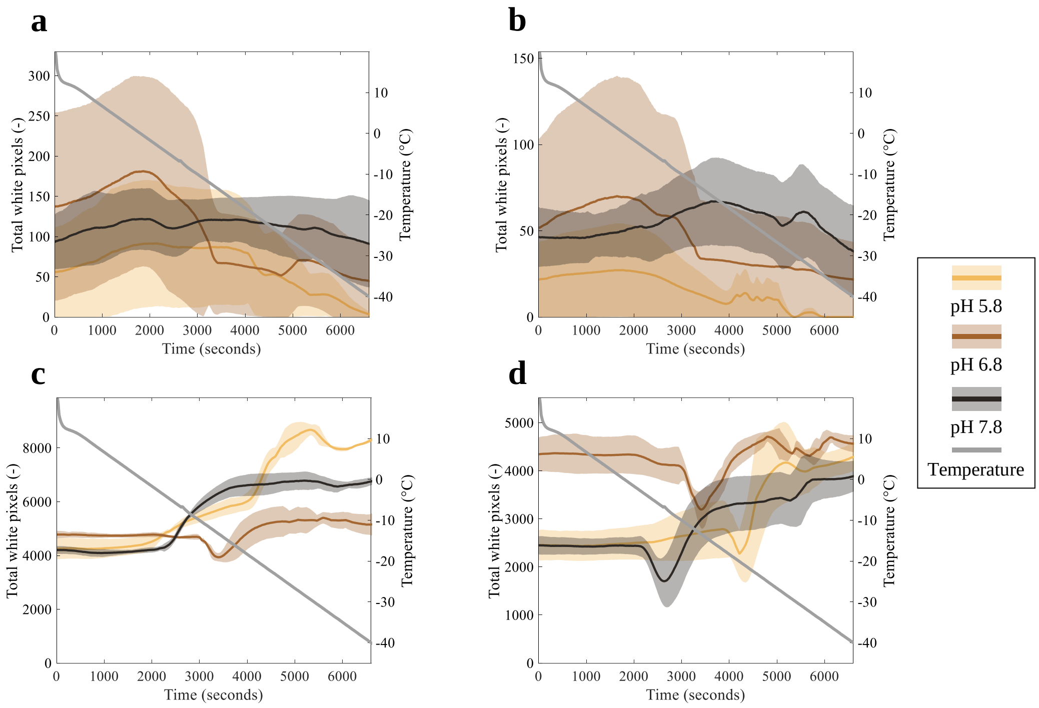

Figure S1: Exemplary results for total white pixels using a white level (a) 0.75, (b) 0.85, (c) mean gray intensity of the first image, and (d) calculated with MATLAB function *graythres* based on the first image (all left y-axis) over time (seconds; x-axis). Each plot shows data for samples at pH 5.8 (yellow), pH 6.8 (brown), and pH 7.8 (gray). The lines indicate the median value and the shades indicate the median absolute deviation of 6 technical replicates, respectively. The temperature (°C) during the measurement is shown on the right y-axis. All data was obtained with a cooling rate of 0.5 °C/min for a sample volume of 20 µL.

To illustrate the information content in the images per image analysis strategy, three images per strategy were extracted. The results are shown in Figure S2.

**Image type**	**White level**	**T = 15 °C**	**$T_{Cloud,app}$**	**T = -40 °C**
Color	-	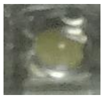	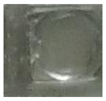	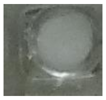
Red	-	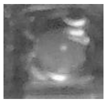	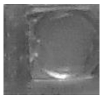	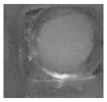
Blue	-	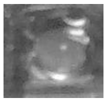	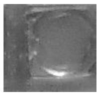	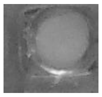
Green	-	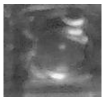	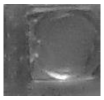	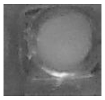
Gray	-	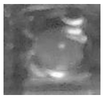	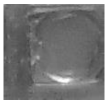	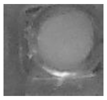
Binary	0.50	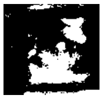	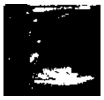	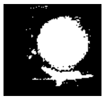
	0.75	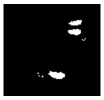	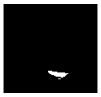	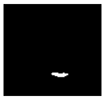
	0.85	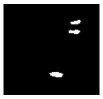	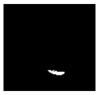	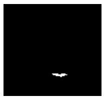
	Intensity	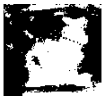	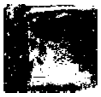	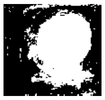
	graythres	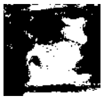	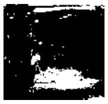	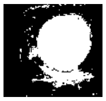

Figure S2: Exemplary images for each image processing approach for first image at 15 ˚C, the apparent protein cloud temperature ($T_{Cloud, app}$), and at -40 ˚C. All images were obtained from a sample with at pH 6.8 measured with a cooling rate of 0.5 °C/min and a sample volume of 20 µL.

The ice nucleation temperature of several demineralized water samples, which were added to each data series, are listed in Table S1.

Table S1: Ice nucleation temperature (°C) of demineralized water samples per data series, given as average ± standard deviation. The number of samples used to calculate the mean and standard deviation is listed in the last column. *n.a.*: not available.

**Cooling rate** (˚C /min)	**Sample volume** (µL)	**Average nucleation temperature** ˚C (± standard deviation)	**Number of samples** -
0.1	24	-31.8 ± 3.7	4
0.2	24	-29.5 ± 3.8	3
0.3	24	-29.7 ± 1.4	2
0.4	24	-27.9 ± *n.a.*	1
0.5	24	-32.8 ± 3.8	4
0.5	20	-31.1 ± 6.0	2
0.5	15	-27.4 ± 3.3	4
0.5	10	-32.1 ± 4.3	4
0.5	5	-34.1 ± 5.1	3

The number of total white pixels, obtained with a white level of 0.50, were also used to extract the apparent protein cloud-point temperature and nucleation temperature for all measured samples. The results are shown in Figure S3. The trends visible in Figure S3 are similar to the results obtained with the total intensity difference image analysis strategy. The absolute values can be found in Table S2 and Table S3.

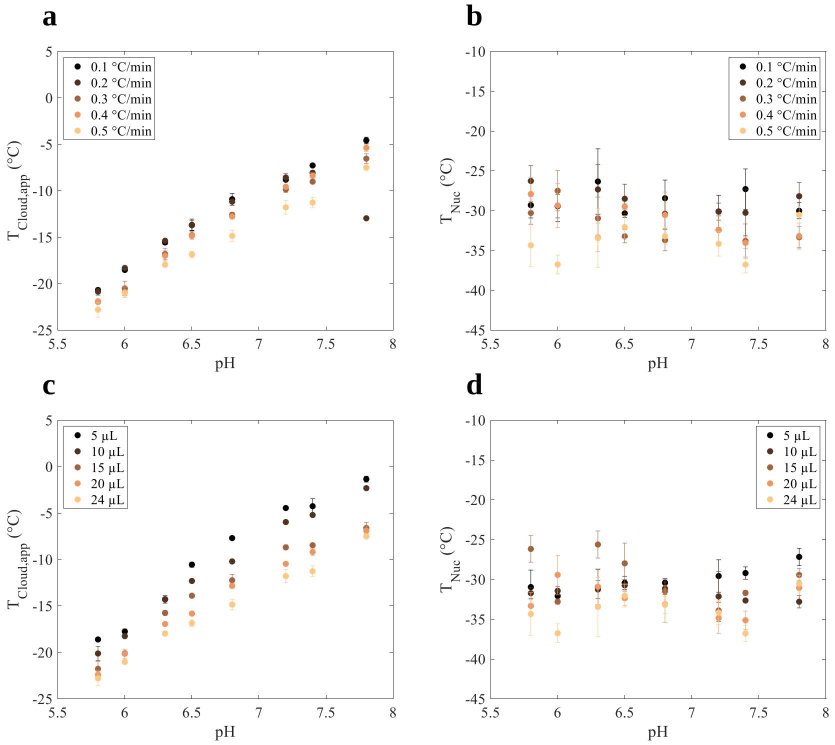

Figure S3: Results obtained with total white pixel image analysis, using a white level of 0.50. (a) Median apparent protein cloud-point temperature ($T_{Cloud,app}$ in °C; y-axis) per pH value (x-axis) for varying cooling rates (0.1 – 0.5 °C/min) and a sample volume of 24 µL, where darker colors indicate slower cooling rates. (b) Median ice nucleation temperature (T_{Nuc} in °C; y-axis) per pH value (x-axis) for varying cooling rates (0.1 – 0.5 °C/min) and a sample volume of 24 µL. Colors are similar to (a). (c) Median $T_{Cloud,app}$ (°C; y-axis) per pH value (x-axis) for varying sample volumes (24 - 5 µL) and a cooling rate of 0.5 °C/min, where darker colors indicate lower sample volumes. (b) Median T_{Nuc} (°C; y-axis) per pH value (x-axis) for varying sample volumes (5 – 24 µL) and a cooling rate of 0.5 °C/min. Colors are similar to (c). All error bars indicate the median absolute deviation.

The absolute values of the ice nucleation temperatures obtained with the total number of white pixels, using a white level of 0.50, are listed in Table S2. These values correspond to the graphs in Figure S3.

Table S2: List of all median ± median absolute deviation (MAD) ice nucleation temperatures (˚C) obtained with total white pixel image analysis, using a white level of 0.50.

Cooling rate (°C/min)	0.1	0.2	0.3	0.4	0.5
pH 5.8	-29.3 ± 1.6	-26.3 ± 1.9	-30.3 ± 1.5	-27.9 ± 2.1	-34.3 ± 2.7
pH 6.0	-29.4 ± 1.9	-29.4 ± 1.5	-27.5 ± 2.5	-29.3 ± 2.8	-36.8 ± 1.2
pH 6.3	-26.3 ± 4.1	-27.3 ± 3.1	-31.0 ± 2.7	-33.4 ± 1.8	-33.4 ± 3.7
pH 6.5	-30.3 ± 0.5	-28.5 ± 1.8	-33.2 ± 0.8	-29.5 ± 0.3	-32.0 ± 1.4
pH 6.8	-28.4 ± 2.3	-30.4 ± 1.9	-33.7 ± 1.3	-30.5 ± 2.9	-33.2 ± 1.1
pH 7.2	-30.1 ± 2.0	-30.1 ± 1.1	-32.4 ± 1.8	-32.4 ± 1.9	-34.2 ± 1.5
pH 7.4	-27.3 ± 2.5	-30.3 ± 2.9	-33.8 ± 2.1	-34.0 ± 0.7	-36.8 ± 1.0
pH 7.8	-30.0 ± 1.0	-28.2 ± 1.7	-33.3 ± 3.1	-33.2 ± 1.6	-30.5 ± 1.1
**Sample volume (µL)**	**5**	**10**	**15**	**20**	**24**
pH 5.8	-31.0 ± 2.1	-31.7 ± 0.7	-26.2 ± 1.7	-33.3 ± 0.8	-34.3 ± 2.7
pH 6.0	-32.1 ± 0.9	-31.4 ± 0.6	-32.8 ± 0.3	-29.4 ± 2.4	-36.8 ± 1.2
pH 6.3	-31.0 ± 2.2	-31.3 ± 1.1	-25.6 ± 1.7	-30.9 ± 2.2	-33.4 ± 3.7
pH 6.5	-30.4 ± 0.8	-30.7 ± 0.7	-28.0 ± 2.5	-32.4 ± 0.9	-32.0 ± 1.4
pH 6.8	-30.4 ± 0.5	-31.2 ± 0.7	-31.5 ± 1.5	-33.1 ± 2.3	-33.2 ± 1.1
pH 7.2	-29.6 ± 2.0	-32.1 ± 3.1	-33.9 ± 1.1	-34.8 ± 1.9	-34.2 ± 1.5
pH 7.4	-29.2 ± 0.8	-32.6 ± 0.2	-31.7 ± 0.3	-35.1 ± 1.2	-36.8 ± 1.0
pH 7.8	-27.2 ± 1.1	-32.8 ± 0.8	-29.4 ± 0.8	-31.1 ± 1.0	-30.5 ± 1.1

Differences in total white pixels (white level 0.50) a protein sample (90 g/L lysozyme at pH 6.5), a blank sample (buffer at pH 6.5), and a demineralized water sample are shown in Figure S4. The data shows that for a buffer solution with and without protein, the total pixel count increases for T_{Nuc}, while the total white pixel count decreases for the demineralized water sample.

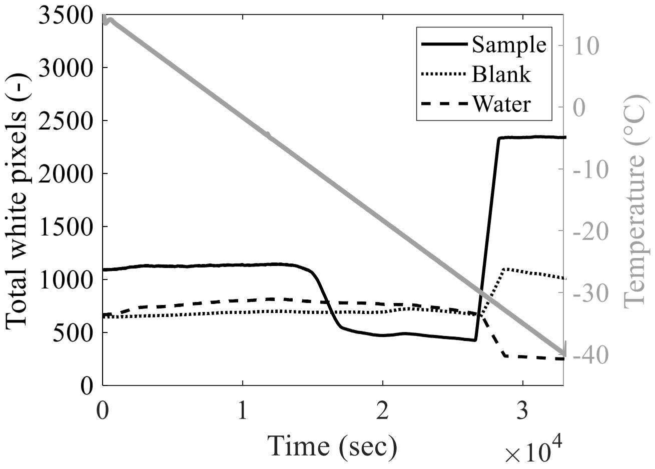

Figure S4: Total white pixel (left y-axis) and temperature (gray; right y-axis) over time (seconds; x-axis) for a protein sample at pH 6.5 (solid), a blank measurement with a buffer solution at pH 6.5 (dotted), and demineralized water (dashed). All data is obtained with a cooling rate of 0.1 °C/min and a sample volume of 24 µL.

The mean red, blue, and green color levels are shown in Figure S5.

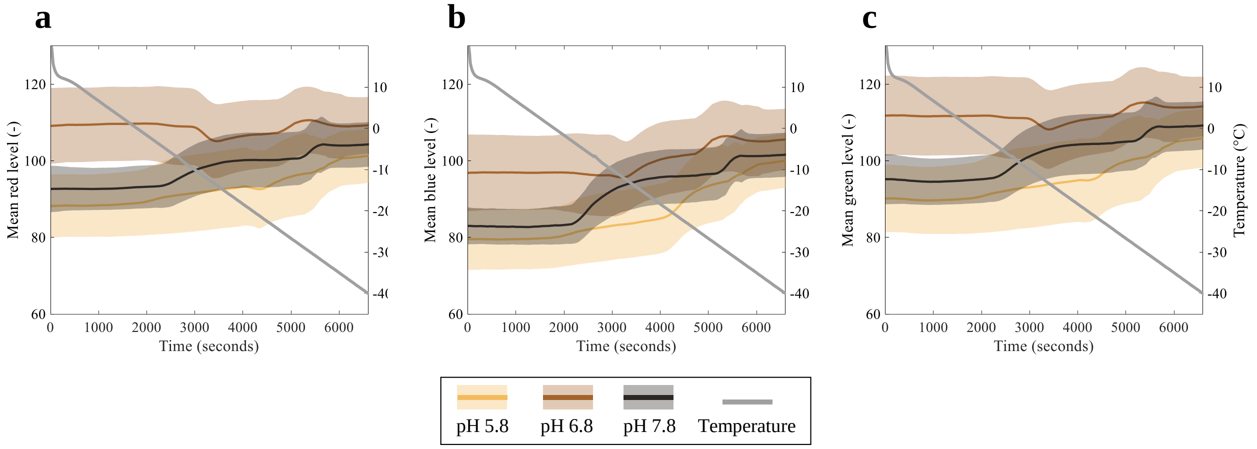

Figure S5: Exemplary results for (a) mean red level, (b) mean blue level, and (c) mean green level (all left y-axis) over time (seconds; x-axis). Each plot shows data for samples at pH 5.8 (yellow), pH 6.8 (brown), and pH 7.8 (gray). The lines indicate the median value and the shades indicate the median absolute deviation of 6 technical replicates, respectively. The temperature (°C) during the measurement is shown on the right y-axis. All data was obtained with a cooling rate of 0.5 °C/min for a sample volume of 20 µL.

One-way ANOVA boxplots for the variance comparison of image analysis strategies TID and TWP, cooling rate (0.1 °C/min and 0.5 °C/min), and sample volume (5 μL and 24 μL) are shown in Figure S6.

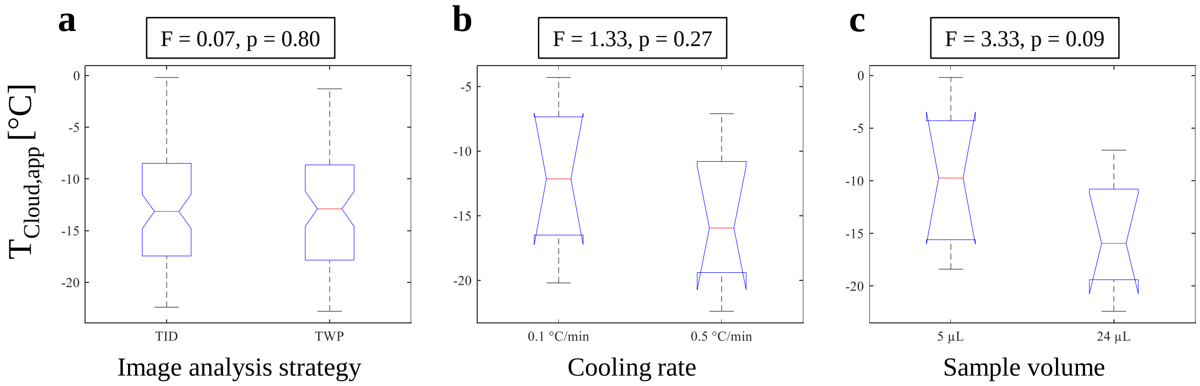

Figure S6: ANOVA boxplots for (a) image analysis strategy (total intensity difference; TID, total number of white pixels; TWP), (b) cooling rate (0.1 °C/min and 0.5 °C/min), and (c) sample volume (5 μL and 24 μL). The corresponding *F*-value and *p*-value are shown above each subplot.

The absolute values of the apparent protein cloud-point temperatures obtained with the total number of white pixels, using a white level of 0.50, are listed in Table S3. These values correspond to the graphs in Figure S3.

Table S3: List of all median ± median absolute deviation (MAD) apparent protein cloud-point temperatures (˚C) obtained with total white pixel image analysis strategy, using a white level of 0.50.

Cooling rate (°C/min)	0.1	0.2	0.3	0.4	0.5
pH 5.8	-20.7 ± 0.2	-20.9 ± 0.4	-21.9 ± 0.2	-21.9 ± 0.0	-22.8 ± 0.8
pH 6.0	-18.5 ± 0.1	-18.3 ± 0.1	-20.5 ± 0.8	-20.9 ± 0.5	-21.0 ± 0.2
pH 6.3	-15.6 ± 0.2	-15.4 ± 0.2	-16.8 ± 0.6	-17.0 ± 0.6	-18.0 ± 0.2
pH 6.5	-13.7 ± 0.6	-13.7 ± 0.5	-14.8 ± 0.4	-14.8 ± 0.1	-16.8 ± 0.3
pH 6.8	-10.9 ± 0.6	-11.2 ± 0.2	-12.6 ± 0.2	-12.8 ± 0.2	-14.8 ± 0.6
pH 7.2	-8.8 ± 0.0	-8.6 ± 0.4	-9.9 ± 0.3	-9.6 ± 0.2	-11.8 ± 0.7
pH 7.4	-7.3 ± 0.1	-8.1 ± 0.2	-9.0 ± 0.0	-8.4 ± 0.3	-11.3 ± 0.6
pH 7.8	-4.6 ± 0.4	-13.0 ± 0.1	-6.5 ± 0.5	-5.4 ± 0.4	-7.5 ± 0.3
**Sample volume (µL)**	**5**	**10**	**15**	**20**	**24**
pH 5.8	-18.6 ± 0.0	-20.1 ± 0.8	-21.8 ± 0.8	-22.4 ± 0.2	-22.8 ± 0.8
pH 6.0	-17.7 ± 0.3	-18.2 ± 0.0	-20.1 ± 0.0	-20.1 ± 0.5	-21.0 ± 0.2
pH 6.3	-14.3 ± 0.4	-14.3 ± 0.0	-15.7 ± 0.3	-16.9 ± 0.1	-18.0 ± 0.2
pH 6.5	-10.6 ± 0.3	-12.3 ± 0.0	-13.9 ± 0.0	-15.8 ± 0.1	-16.8 ± 0.3
pH 6.8	-7.7 ± 0.2	-10.2 ± 0.2	-12.2 ± 0.6	-12.8 ± 0.3	-14.8 ± 0.6
pH 7.2	-4.5 ± 0.1	-6.0 ± 0.1	-8.7 ± 0.2	-10.5 ± 0.1	-11.8 ± 0.7
pH 7.4	-4.3 ± 0.8	-5.2 ± 0.0	-8.5 ± 0.0	-9.2 ± 0.4	-11.3 ± 0.6
pH 7.8	-1.3 ± 0.3	-2.3 ± 0.1	-6.6 ± 0.6	-6.9 ± 0.5	-7.5 ± 0.3

All extracted apparent protein cloud-point temperatures obtained with the total intensity difference image analysis strategy are listed in Table S4.

Table S4: List of all median ± median absolute deviation (MAD) apparent protein cloud temperatures (˚C) obtained with total intensity difference image analysis.

Cooling rate (°C/min)	0.1	0.2	0.3	0.4	0.5
pH 5.8	-20.2 ± 0.1	-20.0 ± 0.1	-19.9 ± 0.3	-21.5 ± 0.2	-22.4 ± 0.6
pH 6.0	-17.9 ± 0.0	-17.5 ± 0.2	-18.9 ± 0.2	-19.4 ± 0.7	-20.7 ± 0.4
pH 6.3	-15.1 ± 0.0	-14.9 ± 0.0	-15.5 ± 0.0	-17.0 ± 0.0	-18.1 ± 0.2
pH 6.5	-13.0 ± 0.0	-13.3 ± 0.1	-14.4 ± 0.1	-15.5 ± 0.2	-17.4 ± 0.1
pH 6.8	-11.3 ± 0.2	-11.2 ± 0.0	-12.5 ± 0.1	-13.6 ± 0.3	-14.5 ± 0.4
pH 7.2	-8.3 ± 0.4	-8.5 ± 0.4	-9.8 ± 0.4	-10.3 ± 0.1	-11.8 ± 0.6
pH 7.4	-6.4 ± 0.4	-7.4 ± 0.6	-8.4 ± 0.5	-8.5 ± 0.3	-9.8 ± 0.4
pH 7.8	-4.3 ± 0.0	-12.0 ± 0.8	-4.5 ± 0.1	-5.1 ± 0.3	-7.1 ± 0.7
**Sample volume (μL)**	**5**	**10**	**15**	**20**	**24**
pH 5.8	-18.4 ± 0.2	-19.3 ± 0.1	-20.5 ± 0.3	-21.7 ± 0.2	-22.4 ± 0.6
pH 6.0	-17.4 ± 0.1	-17.6 ± 0.0	-19.4 ± 0.1	-19.9 ± 0.1	-20.7 ± 0.4
pH 6.3	-13.8 ± 0.3	-14.6 ± 0.3	-16.0 ± 0.1	-16.8 ± 0.3	-18.1 ± 0.2
pH 6.5	-11.1 ± 0.2	-12.8 ± 0.3	-14.1 ± 0.4	-16.4 ± 0.1	-17.4 ± 0.1
pH 6.8	-8.4 ± 0.5	-11.4 ± 0.6	-12.3 ± 0.4	-13.9 ± 0.0	-14.5 ± 0.4
pH 7.2	-4.5 ± 0.2	-7.2 ± 0.4	-9.0 ± 0.2	-10.9 ± 0.6	-11.8 ± 0.6
pH 7.4	-4.1 ± 5.7	-5.7 ± 0.2	-8.7 ± 0.4	-9.7 ± 0.5	-9.8 ± 0.4
pH 7.8	-0.2 ± 1.0	-2.6 ± 0.5	-6.4 ± 0.1	-6.3 ± 0.4	-7.1 ± 0.7
